# Intra-articular gas on knee MRI: a meniscal tear mimic and description of a novel MRI sign

**DOI:** 10.1007/s00256-026-05191-6

**Published:** 2026-03-14

**Authors:** Thomas Saliba, Jacques Malghem, Patrick Omoumi

**Affiliations:** 1 Lausanne University Hospital and University of Lausanne, Lausanne, Switzerland; 2 Cliniques Universitaires St Luc, Brussels, Belgium

**Keywords:** MRI, Knee, Joint, Meniscus, Vacuum

## Abstract

Intra-articular gas is a recognized mimic of meniscal tears on knee MRI, typically resulting from a vacuum phenomenon caused by joint distension and negative intra-articular pressure. This study presents three cases demonstrating transient intra-articular gas that simulated meniscal pathology but was ultimately identified as a benign vacuum phenomenon. We describe a novel diagnostic clue, the “rising tide sign,” which refers to the progressive disappearance of intra-articular gas and the posterior-to-anterior migration of the fluid interface in a gravity-dependent manner, resembling rising tidal water. Recognition of this sign enables differentiation between true meniscal tears and gas artifacts, thereby avoiding false-positive diagnoses and potential unnecessary surgical intervention.

## Introduction

Human joint space pressures are typically sub-atmospheric and are around −5 mmHg [1]. In its resting state, the joint normalizes this pressure by pulling in liquid from the surrounding blood vessels via the synovial microcirculation to create synovial fluid [2]. However, joint distention can create a drop in intra-articular pressure, resulting in the so-called vacuum phenomenon. When this occurs, nitrogen gas accumulates within the joint space and appears as a radiolucent area on radiographs, first described in 1937 by Magnusson [3]. The phenomenon was then described in spine X-rays, where gas from the surrounding tissues accumulates within disk fissures [4]. This phenomenon typically occurs when the joint contains little or no fluid, leading to a negative intra-articular pressure that draws gas from the surrounding extracellular spaces [4].

Ultrasound has also been used to demonstrate the vacuum phenomenon, showing hyperechoic gas bubbles with the joint when traction is applied to the metacarpophalangeal joint, coinciding with the production of a characteristic “cracking sound” [5]. Once this has occurred, the joint enters a refractory period of approximately 20–30 min, during which it cannot be “cracked” again, as the pressure returns to normal and the gas bubbles dissolve and are reabsorbed [5, 6].

This phenomenon is also visible on MRI in degenerative disks, where a hypointense area within the T2-hyperintense disk fluid has been shown to correlate with the vacuum phenomenon on CT scans acquired at a similar time frame [7]. A comparable process occurs in vertebrae affected by avascular necrosis, also known as Kummel disease, where intraosseous gas may be seen on radiographs [4, 5]. Interestingly, MRI correlations showed that the hypointense gas was gradually replaced by fluid, appearing hyperintense on T2-weighted sequences [8]. The presence of intra-articular gas in the context of knee MRIs was first noted in 1991, where it was shown to result in a signal void on T1-weighted imaging and produced susceptibility artifacts on gradient echo sequences (GRE) [9]. GRE sequences, which are highly sensitive to susceptibility artifacts, have largely been supplanted by less artifact-prone FSE imaging for intra-articular disorders in routine clinical practice [9]. However, they were instrumental in demonstrating that intra-articular gas may mimic meniscal tears and thus represent a potential diagnostic pitfall. Although this artifact is relatively rare (1.3% prevalence on 3 T MRI in one study), it is important to be aware of its existence and to differentiate it from true meniscal injury to avoid false positives and unnecessary procedures [10, 11].

Similar to the observations of Malghem et al. in vertebral osteonecrosis, we observed regression of intra-articular gas attributed to a vacuum effect in the knee. This gradual replacement of the intra-articular gas by fluid may help radiologists avoid the common interpretative pitfall of mistaking the gas for a meniscal lesion. We demonstrate this fact with three cases of intra-articular gas mimicking meniscal injuries. Please note that all localizer sequences in the following cases were GRE sequences.

## Case reports

### Case 1

**Fig. 1:**
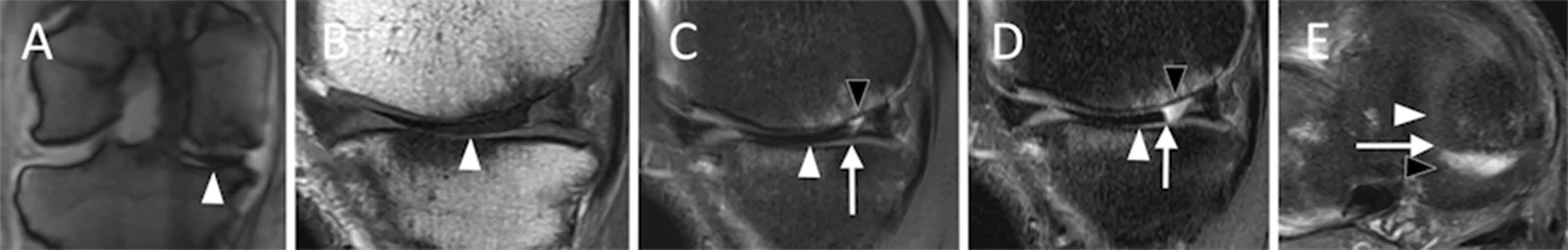
MRI of a 49-year-old man with knee osteoarthritis. **A** Coronal localizer gradient echo image acquired at T = 0 min showing a hypointense linear signal in the articular compartment (white arrowhead), representing gas. **B** T1-weighted sagittal image acquired at T + 2 min showing a hypointense linear signal in the articular compartment (white arrowhead), representing gas. **C** Sagittal T2-map image acquired at T + 12 min showing the area of low signal intensity corresponding to gas (white arrowhead), anterior to intra-articular fluid (black arrowhead), and corresponding fluid-gas interface (white arrow). **D** Sagittal fat-suppressed intermediate-weighted image (FSIW) acquired at T + 19 min showing increasing intra-articular fluid (black arrowhead), replacing the gas (white arrowhead). The fluid-gas interface was displaced anteriorly (white arrow). **E **Axial FSIW image acquired at T + 21 min showing an interface (white arrow) between the intra-articular fluid (black arrowhead) and the gas (white arrowhead), alongside the fluid-gas interface (white arrow)

A 49-year-old man with knee osteoarthritis was referred for an MRI. The scan showed a triangular hypointense structure in the medial femorotibial joint space, with an abrupt vertical transition with the intra-articular fluid posteriorly, mimicking a displaced meniscal fragment (Fig. 1). We suspected that this finding corresponded to intra-articular gas related to a vacuum phenomenon, as evidenced on the localizer gradient echo sequence by susceptibility artefacts within the femorotibial joint space. Successive sequences revealed progressive replacement of the gas by intra-articular fluid over the course of the MRI examination, as the gas bubbles fragmented and dissolved (Fig. 1), definitively proving that the lesion represented gas and not a meniscal fragment. This case demonstrates typical findings of prominent intra-articular gas.

### Case 2

**Fig. 2:**
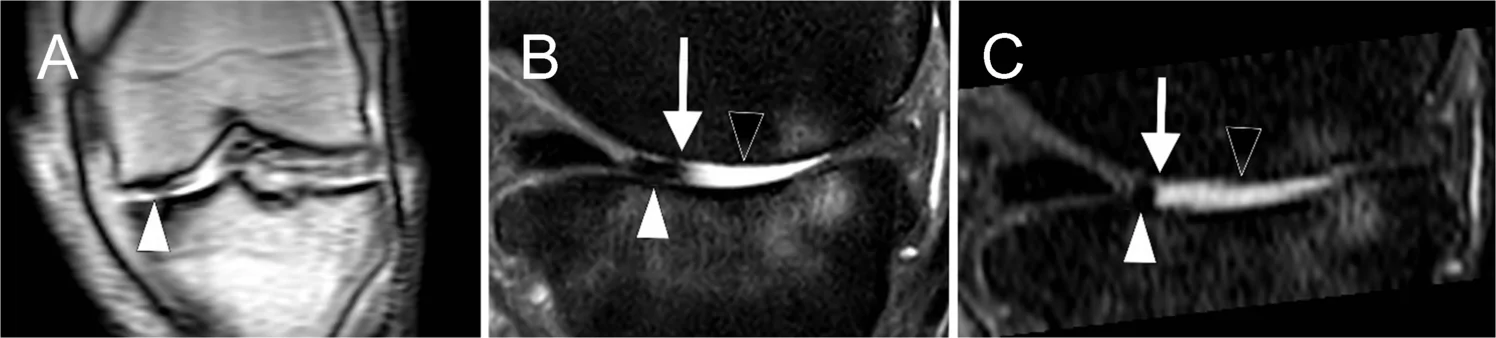
MRI of a 65-year-old man with chronic knee pain. **A** Coronal gradient echo localizer acquired at T = 0 min showing a hypointense linear signal in the articular compartment (white arrowhead), representing gas. **B** Sagittal fat-suppressed intermediate-weighted (FSIW) image acquired at T + 1 min showing a corresponding hypointense area (white arrowhead), which may mimic a meniscal fragment. **C** Sagittal isometric 1-mm FSIW reformat acquired at T + 11 min showing nearly complete replacement of the intra-articular gas (white arrowhead) by intra-articular fluid (black arrowhead) and anterior displacement of the fluid-gas interface (white arrow)

A 65-year-old man presenting with chronic knee pain underwent an MRI. A hypointense structure was seen in the medial compartment, mimicking a meniscal fragment (Fig. 2). However, its appearance and behavior were consistent with intra-articular gas, which could also be seen on the localizer (Fig. 2A). Consecutive sagittal images (Fig. 2B and  2C), showed that the hypointensity diminished in size, and was displaced anteriorly by fluid, confirming that it represented gas rather than a true meniscal fragment.

### Case 3

**Fig. 3:**
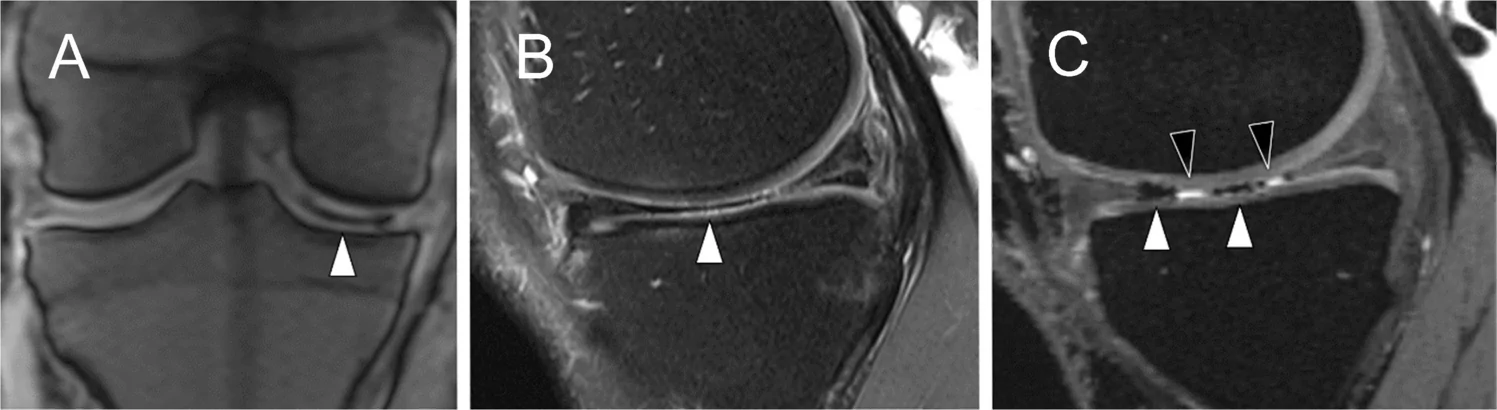
MRI of a 62-year-old asymptomatic man whose examination was performed as part of a research study. **A** Coronal gradient echo localizer acquired at T = 0 min showing intra-articular gas (white arrowhead). **B** Sagittal fat-suppressed intermediate-weighted image (FSIW) acquired at T + 3 min showing a corresponding small hypointense area (white arrowhead), mimicking a meniscal fragment. Note the appearance of irregular cartilage due to the blooming effect of the gas. **C** Sagittal isometric 0.6-mm DESS reconstruction image acquired at T + 41 min showing fragmentation of intra-articular gas (white arrowheads), interspersed with intra-articular fluid (black arrowheads)

A 62-year-old asymptomatic man underwent a knee MRI as part of a research study, which revealed an irregular hypointense structure in the joint space mimicking a meniscal fragment (Fig. 3). However, the marked hypointensity on all sequences, together with the blooming artifact observed on the localizer, was suggestive of intra-articular gas.

During the examination, the hypointense areas were progressively fragmented and displaced by intra-articular fluid (Fig. 3C), confirming that the finding represented gas. There was also a blooming effect on the cartilage surface, which mimicked cartilage pathology. 

## Discussion

Intra-articular gas is a well-recognized mimic of meniscal injury, inducing local magnetic field heterogeneities that cause signal loss and geometric distortion (susceptibility artifacts). It may appear as an area of low signal intensity on FSE sequences, including T1, T2, and intermediate-weighted FSE imaging, and produce susceptibility artifacts on GRE MR sequences [9]. This artifact’s signal intensity and shape may be similar to meniscal tissue and simulate a displaced meniscal fragment or tear, potentially leading to misinterpretation [9]. Because magnetic field inhomogeneities increase with field strength, the susceptibility artifact from intra-articular gas is greater at 3 T than at 1.5 T, making its recognition increasingly important as 3 T MRI systems become more prevalent [12].

Occasionally, intra-articular gas may be visible on radiographs obtained before or after the MRI examination. However, it can be absent if the radiograph is performed before the vacuum phenomenon develops. It can also be missed on radiographs, as small amounts of gas may remain undetectable with this modality [5, 13]. GRE sequences can provide a diagnostic clue because of their sensitivity to susceptibility artifacts [13]. However, intermediate-weighted FSE sequences have largely replaced GRE sequences for evaluating cartilage and internal derangement of joints. Therefore, GRE sequences may be absent from modern knee MRI protocols and thus can no longer be consistently relied upon [14]. GRE-based localizers are commonly used and may reveal the presence of gas; however, because they are designed for rapid acquisition, they may lack the spatial resolution necessary to reliably detect small gas collections.

Another method to differentiate intra-articular gas from meniscal injury was proposed by Sakamoto et al. and relies on a 3D SPACE sequence on 3 T imaging [10]. Using this sequence, they suggested that one could distinguish gas from meniscal tissue by relying on the high spatial resolution of the sequence to see the gas conforming to the irregular surfaces of the cartilage [10]. However, the irregularities described on the cartilage surface may also be related to blooming artifacts due to the presence of gas in the joint space, rather than gas filling voids in the cartilage. Furthermore, the authors observed that this method is less reliable than detecting susceptibility artifacts on GRE localizers for confirming the presence of gas [10].

This case series illustrates an additional method to differentiate intra-articular gas from displaced meniscal fragments/meniscal tears: monitoring the progressive shift of the gas–fluid interface as gas is replaced by fluid on consecutive MRI sequences. This finding can be corroborated by reviewing the time stamps of each sequence, which help confirm the temporal progression of gas replacement by intra-articular fluid. In observing this sign, it is important to remember that the patient lies supine on the scanning table during image acquisition, with the knee extended. This position explains why gas is typically located anteriorly compared to fluid, which is pulled downward (posteriorly) by gravity due to it higher density. Consequently, the replacement of gas by fluid progresses from the posterior to the anterior part of the knee in a gravity-dependent manner. This gradual posterior-to-anterior shift of the gas–fluid interface can be described as the “rising tide” sign. Furthermore, this sign has the advantage of being MRI field strength independent and visible on any sequence weighting, although best seen on fluid-sensitive sequences. Therefore, in the absence of dedicated GRE sequences, this sign can provide assurance that what is seen is indeed gas and not a meniscal fragment.

## Conclusion

Intra-articular gas forms when the joint is distracted and negative pressure within the joint space creates a vacuum effect. Intra-articular gas can mimic a displaced meniscal fragment or meniscal tear, and as such it is important to be able to differentiate it from true pathology. We describe the “rising tide sign,” in which sequential analysis of consecutive sequences demonstrates the gradual displacement and eventual disappearance of intra-articular gas as synovial fluid refills the joint space. This sign may be particularly useful when gradient-echo–based localizers are unavailable or lack the spatial resolution to demonstrate the susceptibility effects associated with intra-articular gas.

## Data Availability

N/A.
